# Salinomycin effectively eliminates cancer stem-like cells and obviates hepatic metastasis in uveal melanoma

**DOI:** 10.1186/s12943-019-1068-1

**Published:** 2019-11-13

**Authors:** Jingfeng Zhou, Shenglan Liu, Yun Wang, Wei Dai, Hailin Zou, Shubo Wang, Jing Zhang, Jingxuan Pan

**Affiliations:** State Key Laboratory of Ophthalmology, Zhongshan Ophthalmic Center, Sun Yat-sen University, 54 South Xianlie Road, Guangzhou, 510060 People’s Republic of China

**Keywords:** Uveal melanoma, Salinomycin, Twist1, Cancer stem-like cells, Metastasis

## Abstract

**Background:**

Uveal melanoma (UM) is the most common primary intraocular tumor. Hepatic metastasis is the major and direct death-related reason in UM patients. Given that cancer stem-like cells (CSCs) are roots of metastasis, targeting CSCs may be a promising strategy to overcome hepatic metastasis in UM. Salinomycin, which has been identified as a selective inhibitor of CSCs in multiple types of cancer, may be an attractive agent against CSCs thereby restrain hepatic metastasis in UM. The objective of the study is to explore the antitumor activity of salinomycin against UM and clarify its underlying mechanism.

**Methods:**

UM cells were treated with salinomycin, and its effects on cell proliferation, apoptosis, migration, invasion, CSCs population, and the related signal transduction pathways were determined. The in vivo antitumor activity of salinomycin was evaluated in the NOD/SCID UM xenograft model and intrasplenic transplantation liver metastasis mouse model.

**Results:**

We found that salinomycin remarkably obviated growth and survival in UM cell lines and in a UM xenograft mouse model. Meanwhile, salinomycin significantly eliminated CSCs and efficiently hampered hepatic metastasis in UM liver metastasis mouse model. Mechanistically, Twist1 was fundamental for the salinomycin-enabled CSCs elimination and migration/invasion blockage in UM cells.

**Conclusions:**

Our findings suggest that targeting UM CSCs by salinomycin is a promising therapeutic strategy to hamper hepatic metastasis in UM. These results provide the first pre-clinical evidence for further testing of salinomycin for its antitumor efficacy in UM patients with hepatic metastasis.

## Background

Uveal melanoma (UM) is the most common primary intraocular tumor that originates from melanocytes of eye uveal tract [1]. Although multimodal therapeutic approaches including enucleation, radiotherapy and phototherapy can sufficiently manage localized tumors [2], approximately 50% patients with UM manifest distant organ metastasis, predominantly to the life-sustaining organ liver, even the primary tumors have been removed by enucleation [3, 4]. Hepatic metastasis is among the major and direct death-related reasons in these UM patients [5].

The past decade witnessed an advance in pathophysiology of UM, discovering that exclusive mutations in GNAQ and GNA11 observed in ~ 80% of patients with UM can drive the growth of the primary UM lesions [6, 7]. Agents targeting protein kinase C (PKC) and mitogen-activated protein kinase (MAPK) which are downstream effector molecules of such G protein coupled receptor (GPCR) proteins were evaluated in clinical trials, somehow showing disappointing results [8, 9]. Similarly, whether the ongoing clinical trial regard targeting membrane receptor tyrosine kinase c-Met on UM cells can provoke improved survival rate in patients with hepatic metastasis is not clear [8, 10]. Therefore, the development of innovative and effective drugs for hepatic metastasis in UM still has a high priority.

Cancer stem-like cells (CSCs) are a subpopulation cells in the cancerous tissue which are featured with self-renewal, differentiation into bulk tumor cells, quiescence, and drug-resistance [11–16]. CSCs are therefore believed roots of metastasis, drug-resistance and relapse [14]. It is plausible that targeting CSCs is critical for management of metastasis.

Salinomycin, an approved agricultural antibiotic to prevent coccidiosis in poultry for decades, has re-attracted attention in the bio-medical fields since it was identified as a selective inhibitor of CSCs in a variety of types of cancer (e.g., breast cancer, leukemia and colorectal cancer) [17–20]. The relevant anti-CSCs mechanisms of salinomycin involve in inhibition of stemness-associated transcriptional factors such as β-catenin, c-Myc, Snail and SOX2 [17, 21–26]. In the present study, we envisaged that salinomycin might be capable of eradicating CSCs and thereby hampering hepatic metastasis in UM. We interrogated this hypothesis and found that salinomycin perturbed traits of CSCs and obviated hepatic metastasis in UM. Mechanistically, Twist1 was fundamental for the salinomycin-enabled CSCs elimination and migration/invasion blockage in UM cells. These results indicate that salinomycin might be a promising agent for the treatment of UM patients with hepatic metastasis.

## Materials and methods

### Chemicals

**Fig. 1:**
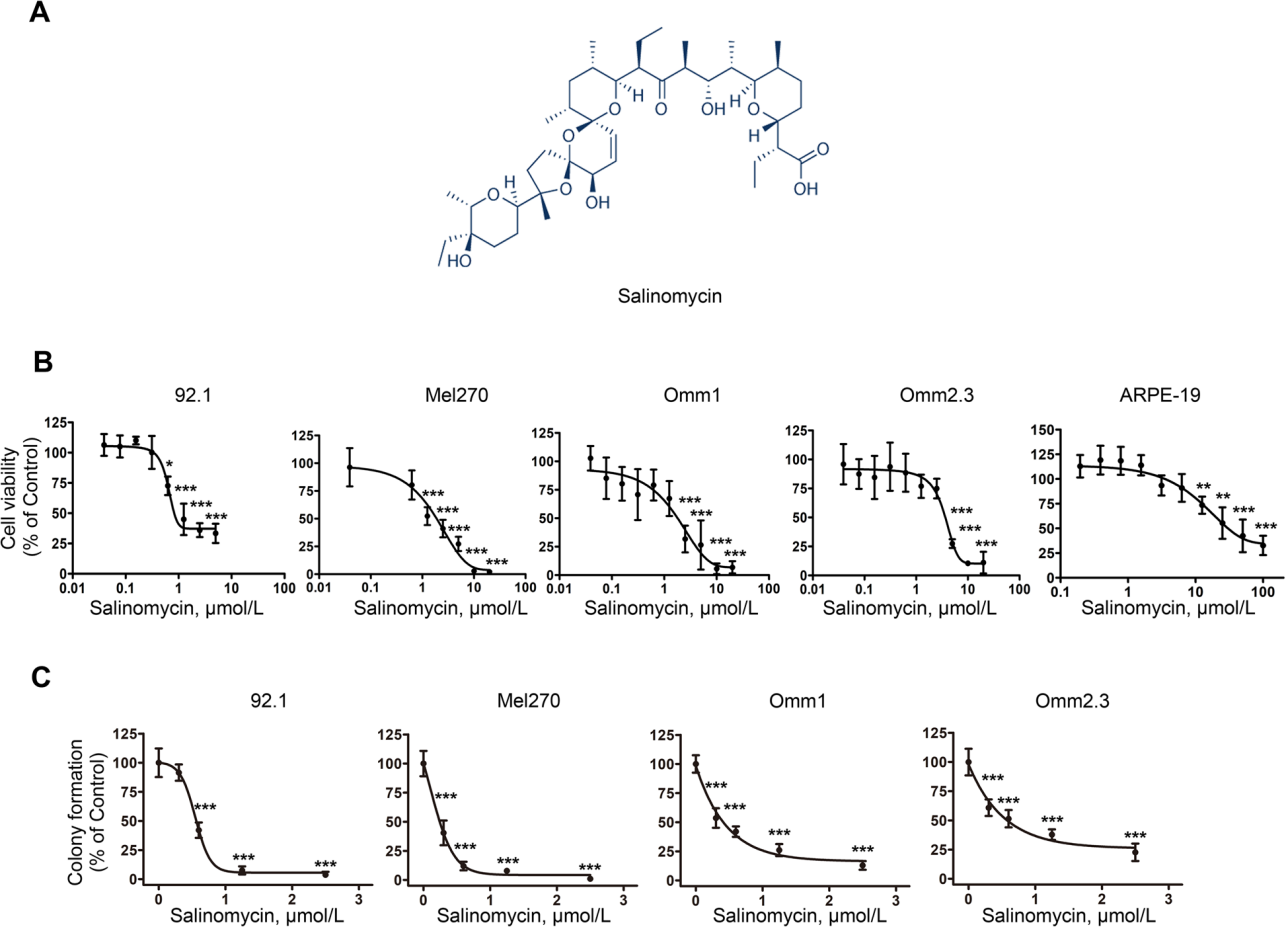
Salinomycin counteracts the proliferation of uveal melanoma cells. **a** Chemical structure of salinomycin. **b** Uveal melanoma (UM) cells and ARPE-19 cells were treated with a gradient concentrations of salinomycin for 72 h, and the cell viability was measured by MTS assay. Data represent mean ± SD. *, *P* < 0.05; ***, *P* < 0.001, one-way ANOVA, post hoc comparisons, Tukey’s test. **c** After UM cells were treated with the indicated concentrations of salinomycin for 24 h, viable cells were seeded into drug-free agarose-containing culture for 14 days. Clonogenicity was expressed by normalizing to control in colony count. Data represent mean ± SD. ***, *P* < 0.001, one-way ANOVA, post hoc comparisons, Tukey’s test

Salinomycin (Fig. 1a) was from MedChemExpress (Shanghai, China), Annexin V-FITC, propidium iodide (PI) were from Sigma-Aldrich (Shanghai, China). Salinomycin was dissolved in DMSO at 20 mmol/L and stored at − 20 °C.

### Cell lines and culture

The human UM cells 92.1, Mel270, Omm1, and Omm2.3 were grown in RPMI1640 medium (Invitrogen, Shanghai, China) supplemented with 10% (*v/v*) fetal bovine serum (Hyclone, Guangzhou, China), 100 U/ml penicillin and 0.1 mg/ml streptomycin as described previously [27]. The cells were incubated at 37 °C humidified incubator in a 5% CO_2_ atmosphere. The cells were routinely examined to ensure mycoplasma-free. All cells used in the experiment were at the logarithmic growth phase.

### Cell viability assay

Cell viability was measured using MTS assay (CellTiter 96 Aqueous One Solution reagent, Promega, Beijing China) as described previously [28]. Briefly, UM cells were seeded into a 96-well plates overnight at a density of 5000 cells per well. Salinomycin was added in quadruplicates per concentration (ranging from 0.039 μmol/L to 20 μmol/L with equal ratio dilution approach) for each cell line and incubated for 72 h. At the end of treatment, MTS (20 μL/well) was added to each well, and optical density was read with a wave length of 490 nm. Cell viability was calculated using the following formula: Relative viability = (Mean absorbance of treated wells − background absorbance) / (Mean absorbance of untreated wells − background absorbance) × 100%. The half-maximal inhibitory concentration (IC_50_) of salinomycin at which the cell viability was declined by 50% relative to the untreated control was determined by curve fitting of the sigmoidal dose-response curve GraphPad Prism 5 software (GraphPad, San Diego, CA).

### Colony-formation assay

The colony-forming capacity of UM cells was determined by use of double-layer agarose system as described previously [28]. Briefly, 24-well plates were pre-coated with 400 μL of complete medium containing 1% agarose (Invitrogen, Shanghai, China). Then, UM cells pretreated with different concentrations (0, 1, 2, 4 μmol/L) of salinomycin for 24 h were washed, and seeded over the layer of the drug-free agarose-containing medium in triplicate at a density of 5000 cells/well. After solidification at 4 °C for 10 min, theses plates were incubated at 37 °C in a humidified atmosphere containing 5% CO_2_ for 2 weeks. The colonies consisting of ≥50 cells were then counted under an inverted phase-contrast microscope.

### Apoptosis assay

Apoptosis was assessed using Annexin V- fluoresceinisothiocyanate (FITC) / PI double staining kit (Sigma-Aldrich, Shanghai) according to the manufacturer’s instructions. Briefly, UM cells (2 × 10^5^ cells per well) were seeded overnight into a 6-well plates and treated with salinomycin at a gradient concentrations (0, 2.5, 5, 10 μmol/L) for 24 h or at the indicated time. After treatment, UM cells were harvested and stained with Annexin V-FITC for 15 min at room temperature in dark. PI was added to distinguish the living from the dead cells. Apoptosis was analyzed by using a FACS LSRFortessa flow cytometer with its software [27].

### Western blot analysis

Whole cell lysates were prepared in radioimmunoprecipitation assay (RIPA) buffer (1 × PBS, 1% NP-40, 0.5% sodium deoxycholate, and 0.1% SDS) supplemented with freshly added 10 mmol/L β-glycerophosphate, 1 mmol/L sodium orthovanadate, 10 mmol/L NaF, 1 mmol/L phenylmethylsulfonyl fluoride, and 1 × Roche complete Mini protease inhibitor cocktail (Roche, Indianapolis, IN) [29].

Cytosolic fraction for cytochrome c detection was prepared using the digitonin extraction buffer (10 mmol/L PIPES, 0.015% digitonin, 300 mmol/L sucrose, 100 mmol/L NaCl, 3 mmol/L MgCl_2_, 5 mmol/L EDTA, and 1 mmo/L phenylmethylsulfonyl fluroride) [30]. Briefly, cell pellets were suspended in digitonin extraction buffer on ice for 10 min. Cells were then centrifuged at 14, 000 rpm for 10 min, and the supernatant was transferred to a fresh tube. The resultant supernatant is the cytosolic fraction, and the mitochondria-containing pellets were washed three times, and lysed in RIPA buffer.

Thirty micrograms of cellular proteins were loaded in each well and were separated by sodium dodecyl sulfate polyacrylamide gel electrophoresis (SDS-PAGE). Antibodies against caspase-3 (1 μg, Cat#: 610322), PARP (clone 4C10–5, 1 μg, Cat#: 551024), XIAP (1 μg, Cat#: 610762), Cytochrome c (clone 6H2.B4, 2 μg, Cat#: 556432), β-catenin (2 μg, Cat#: 610154), ALDH (1 μg, Cat#: 611194) were obtained from BD Biosciences (San Jose, CA). Antibodies against Bcl-X_L_ (1.15 μg, Cat#: 2764S), PTEN (0.028 μg, Cat#: 9188S), OCT4 (0.28 μg, Cat#: 2750S), SOX2 (0.096 μg, Cat#: 2750S), Snail (0.44 μg, Cat#: 3879), Twist1 (0.45 μg, Cat#: 46702) and active caspase-3 (0.2 μg, Cat#: 9661S) were purchased from Cell Signaling Technology (Beverly, M, A). Antibody against β-actin (2 μg, Cat#: A5441) was from Sigma-Aldrich (Shanghai, China). Antibody against Bcl-2 (clone 100**,** 4 μg, Cat#: 05–729) was from MilliporeSigma (Danvers, MA). Antibodies against KLF4 (4 μg, Cat#: NBP2–24749), and Survivin (4 μg, Cat#: NB500–201) were from Novus Biologicals (Littleton, CO). Immunoglobulin G fluorescent-conjugated secondary antibodies anti-mouse (0.5 μg, Cat#: 926–32,210) and anti-rabbit (0.5 μg, Cat#: 926–32,211) were from LI-COR Biotechnology (Nebraska, USA). The NC membranes were scanned using the Odyssey infrared imaging system (LI-COR).

### Measurement of mitochondrial transmembrane potential

Salinomycin-induced loss of the inner mitochondrial transmembrane potential (△Ψm) were measured using the MitoTracker probes (CMXRos and MTGreen, Invitrogen) as described previously [28, 30]. Briefly, after treatment with 10 μmol/L salinomycin, the UM cells were harvested and incubated with CMXRos (300 nmol/L) and MTGreen (100 nmol/L) at 37 °C for 1 h in dark. The cells were then washed with PBS and resuspended in serum-free RPMI1640 medium and cellular fluorescence was detected by using a FACS LSRFortessa flow cytometry as described previously [27].

### cDNA constructs and transduction in UM cells

Human baculoviral IAP repeat containing 5 (*BIRC5*, NCBI Reference Sequence ID: NM_001168) were cloned into the pTSB-CMV-MCS-SBP-RFP-F2A-PuroR (lentivirus) construct (Transheep, Shanghai, China) using ClonExpress MultiS One Step Cloning Kit (Vazyme, Nanjing, China). pBABE-puro-mTwist was purchased from Addgene (plasmid # 1783) [31]. Scramble (pLKO.1-puro-Non-target shRNA), human *BIRC5* specific target shRNA (pLKO.1-puro-h*BIRC5*-target shRNA) and human *TWIST1* specific target shRNA (pLKO.1-puro-h*TWIST1*-target shRNA) were purchased from Sigma-Aldrich (Shanghai, China). The lentivirus and retrovirus supernatants were prepared by 293 T cells using the lentivirus packing system [pCMV-dR8.2 (the packing construct), the pCMV-VSVG (envelope construct)] and the PCL retrovirus packing system, respectively [27, 31]. In brief, the lentivirus or retrovirus plasmids together with its corresponding packing constructs were transfected into 293 T cells using polyethyleneimine (Polysciences, Inc., Warrington, PA). Forty-eight or 72 h after transfection, virus-containing supernatants were harvested and purified with 0.45 μm filter. Medium containing lentivirus or retrovirus and polybrene (8 μg/mL) was added to the culture of UM cells. After two rounds of infection, the transduced UM cells were then treated with puromycin (1 μg/mL) for approximately 5 days to establish clones to stably overexpress or silence the corresponding target gene.

### Real-time quantitative PCR (qRT-PCR) analysis

Total RNA isolation was performed with Trizol reagent (Invitrogen) as described previously [30]. The quality and concentration of RNA was assessed by NanoDrop- 2000 spectrophotometer (Thermo Fisher, Shanghai, China) and then 500 ng of total RNA was reverse-transcribed into cDNA with the Maxima First Strand cDNA Synthesis Kit (Thermo Fisher) in a 20 μL reaction volume as described previously [30]. One hundred nanograms cDNA was used for qRT-PCR using SYBR Premix Ex Taq II (Takara Biomedical Technology, Beijing, China) and gene-specific primer sets. GAPDH was used as an internal control. The relative expression of genes were normalized to GAPDH, by using 2^−ΔΔCt^ method, where ΔΔCt = (C_t target gene_ - C_t GAPDH_) treated cells - (C_t target gene_ - C_t GAPDH_) control cells. The primers are listed as follows. *BIRC5*: (F) CATCTCTACATTCAAGAACTGG-3, (R) GGTTAATTCTTCAAACTGCTTC [32]; *TWIST1*: (F) TAGATGTCATTGTTTCCA GAGAAGG, (R) ATTTCCAAGAAAATCTTTGGCA. GAPDH: (F) GCAAATTCCATGGCACCGTC, (R) TCGCCCCACTTGATTTTGG.

### Xenograft experiments

Three million of Omm1 cells were subcutaneously injected into the flanks of 4- to 6-week male NOD/SCID mice (Vitalriver, Beijing, China). Approximately 3 weeks after subcutaneous injection, palpable tumor appeared. Tumors were measured with calipers every other day and tumor volumes were calculated using the following formula: *a*^2^ × *b* × 0.4, where *a* refers to the smallest diameter and *b* is the diameter perpendicular to *a*. When the tumors reached ~ 50 mm^3^, tumor-bearing mice were randomized into two groups (8 mice per each group) and administrated with vehicle (corn oil) or salinomycin (*i.p.*, 2.5 mg/kg, QD) for 21 days. After the mice were euthanized, the tumor xenografts were dissected, weighed and detected in paraffin-embedded sections stained with haematoxylin–eosin (H&E), Ki67, Active-caspase-3 or Twist1. The animal studies were approved by the Sun Yat-sen University Institutional Animal Care and Use Committee.

### Wound-healing scratch assay

The wound-healing scratch assay was performed as described previously [28]. Briefly, UM cells (5 × 10^5^ cells per well) were seeded into 6-well plates and grown to confluence. A straight line scratch was created using a sterile 200 μL disposable micropipette tip. The debris were removed by washing with PBS, and replaced with RPMI1640 medium. The cells were then treated with salinomycin (1.25 μmol/L) and the same microscopic scratch wound area was photographed using an inverted phase-contrast microscope at indicated time.

### Transwell migration and invasion assay

The transwell migration and invasion assays were conducted as previously described [27]. Briefly, UM cells were pre-incubated with 1.25 μmol/L salinomycin for 24 h prior to seeding. Then, the cells were trypsinized, washed with PBS and counted by trypan blue exclusion assay. For migration assay, a total of 5000 viable cells in 200 μL serum-free RPMI1640 medium were seeded into the upper chamber (inserts); for invasion assay, 4 × 10^4^ viable cells were seeded in the upper chamber that pre-coated with matrigel (BD Biosciences, San Jose, CA). The lower chambers were filled with RPMI1640 medium containing 10% FBS to serve as a chemo-attractant. After forty-eight hours, the upper surface was wiped away with a cotton swab to remove the non-migrated cells. The cells migrated or invaded into the bottom of the inserts were then fixed in 3% paraformaldehyde followed by staining with 0.5% crystal violet. After drying, the cells in 3 random microscopic fields were photographed and counted using an inverted phase-contrast microscope.

### Hepatic metastasis mouse model

A hepatic metastasis mouse model via intrasplenic transplantation of UM cells was performed as described [27]. In brief, 5 × 10^5^ Mel270-luciferase cells were intrasplenically injected into the 4- to 6-week male NOD/Shi-scid/IL-2Rγnull (NOG) mice (Vitalriver, Beijing, China) under anesthesia. The mice were randomized into two groups (4 mice in each group) and administrated with vehicle (corn oil) or salinomycin (*i.p.*, 2.5 mg/kg, QD) for 21 days. In vivo bioluminescence imaging of hepatic metastasis was performed after mice were anesthetized with isoflurane using the IVIS Lumina II (Perkin Elmers). The animal studies were approved by the Sun Yat-sen University Institutional Animal Care and Use Committee.

### Melanosphere culture

Melanosphere formation assays were performed as described [28]. Twenty-four hours after treated with salinomycin (2.5 μmol/L), UM cells were harvested, washed with PBS and grown in ultralow-attachment 24-well plates (Thermo Fisher, Shanghai, China) at a density of 5000 cell/well in DMEM/F12 medium (HyClone, containing B27: 1 mL, bFGF: 10 ng/mL and EGF: 20 ng/mL). Seven days later, the melanospheres were harvested, dissociated to single cells and re-plated (5000 cells/well) for the secondary and tertiary rounds of melanosphere formation, respectively. The melanosphere numbers (cells ≥50) were counted under a microscope on day 7 following each round of re-plating [27].

### Aldehyde dehydrogenase assay

Aldehyde dehydrogenase (ALDH) was carried out using the ALDEFLUOR™ kit (Stem Cell Technologies, Vancouver, BC, Canada) following the manufacturer’s guidelines. Briefly, UM cells were treated with 1.25 μmol/L salinomycin for 24 h and suspended in ALDEFLUOR™ assay buffer containing ALDEFLUOR™ reagent (Bodipy-Aminoacetaldehyde) in the presence or absence of the ALDEFLUOR™ DEAB reagent (a specific ALDH1 enzyme inhibitor, work as a reference control). After incubation at 37 °C for 45 min, the cells were washed, resuspended in ALDEFLUOR™ assay buffer and then analyzed by FACS LSRFortessa flow cytometer [33].

### Uveal melanoma survival analysis

Kaplan–Meier survival analyses for metastasis-free survival and overall survival in patients with UM were performed using web tool PROGgene V2 (http://watson.compbio.iupui.edu/chirayu/proggene/database/?url=proggene) [34]. The UM patients were divided into two subgroups on the basis of the median expression level of *SOX2* and *TWIST1*.

### Statistical analysis

All in vitro experiments were repeated three times and data were expressed as mean ± standard deviation (SD). Comparisons between 2 groups were analyzed by 2-tailed Student’s *t* test; differences among multiple groups were analyzed by one-way ANOVA with post hoc comparison by the Tukey’s test, unless otherwise stated. GraphPad Prism 5 software was used for statistical analysis. *P* < 0.05 was considered statistically significant.

## Results

### Salinomycin restricts cellular growth of UM cells

We first evaluated the effect of salinomycin on cell viability of UM cells. MTS assay showed that salinomycin effectively dampened cell viability of 92.1, Mel270, Omm1, and Omm2.3 cells, with IC_50_ values of 1.79 μmol/L, 2.37 μmol/L, 1.77 μmol/L, 3.6 μmol/L, respectively (Fig. 1b). In contrast, salinomycin displayed 11~21-fold less cytotoxicity in normal retinal pigmented epithelium cells (ARPE-19) with IC_50_ value of 38.08 μmol/L (Fig. 1b). Meanwhile, UM cells incubated with salinomycin exhibited marked retardation in clonogenicity as measured in agarose-containing culture with IC_50_ values of 0.67 μmol/L, 0.31 μmol/L, 0.64 μmol/L, 0.56 μmol/L in 92.1, Mel270, Omm1, and Omm2.3 cells, respectively (Fig. 1c). Collectively, these data reveals that salinomycin effectively inhibits growth of UM cells with low cytotoxicity in vitro.

### Salinomycin induces apoptosis in UM cells

**Fig. 2:**
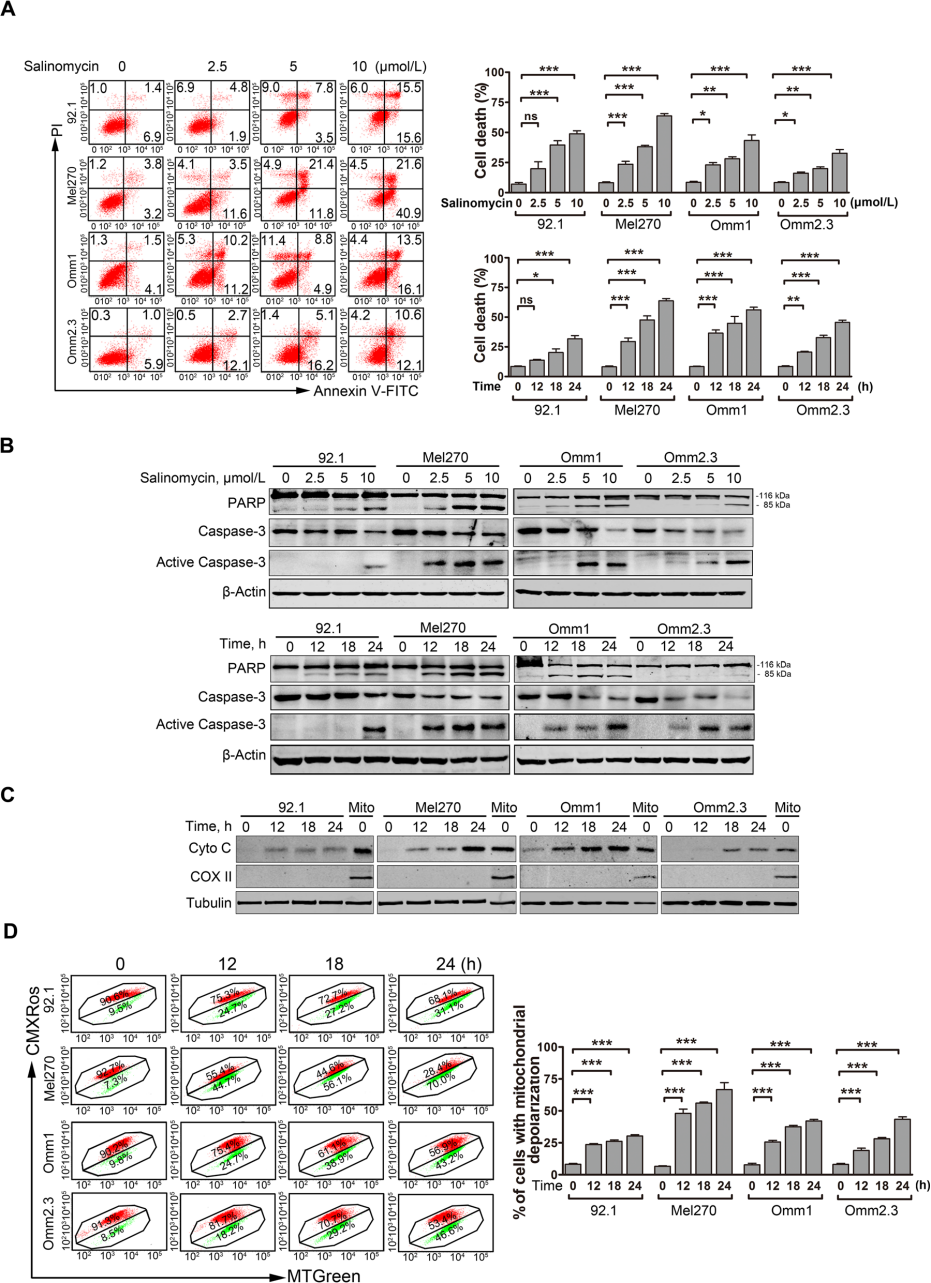
Salinomycin elicits apoptosis in UM cells. **a** Annexin V/PI apoptotic assay was performed in the UM cells treated with escalating concentrations of salinomycin for 24 h or at 10 μmol/L for various exposure time. Representative flow cytometry dot plots *(left*) for UM cells and quantitative analysis (*right*) from three independent experiments are shown. Data represent mean ± SD. ns, not significant; *, *P* < 0.05; **, *P* < 0.01; ***, *P* < 0.001, one-way ANOVA, post hoc comparisons, Tukey’s test. **b** Dose- and time-dependent of apoptosis-specific cleavage of PARP and caspase-3 activation was measured by Western blot after UM cells were incubated with increasing concentrations of salinomycin for 24 h or at 10 μmol/L for the indicated time periods. **c** UM cells were treated with 10 μmol/L salinomycin for different time, the levels of cytochrome c in the cytosolic extracts were analyzed by Western blot. COX II served as a mitochondrial content indicator. **d** UM cells were treated with 10 μmol/L salinomycin for the time indicated, and the mitochondrial potential was then detected by flow cytometry after dual staining with CMXRos and MTGreen. Data represent mean ± SD. ***, *P* < 0.001, one-way ANOVA, post hoc comparisons, Tukey’s test

We next evaluated the capability of salinomycin to induce apoptosis. Salinomycin displayed a remarkable apoptosis-inducing ability in a dose- and time-dependent fashion in UM cells as determined by Annexin V/PI dual staining assay (Fig. 2a). As well, salinomycin induced a concentration- and time-dependent specific cleavage of poly (ADP-ribose) polymerase (PARP) and activation of caspase-3 in UM cells (Fig. 2b). In addition, salinomycin time-dependently elicited accumulation of cytochrome c in cytosol as detected by Western blot analysis (Fig. 2c). Because loss of mitochondrial transmembrane potential (ΔΨm) is a feature of apoptosis, we next evaluated whether salinomycin damaged mitochondria. We found that the proportion of UM cells with loss of ΔΨm was substantially increased with salinomycin treatment as measured by flow cytometric analysis of CMXRos and MTGreen dual staining (Fig. 2d).

### Survivin is protective in salinomycin-mediated apoptosis in UM cells

**Fig. 3:**
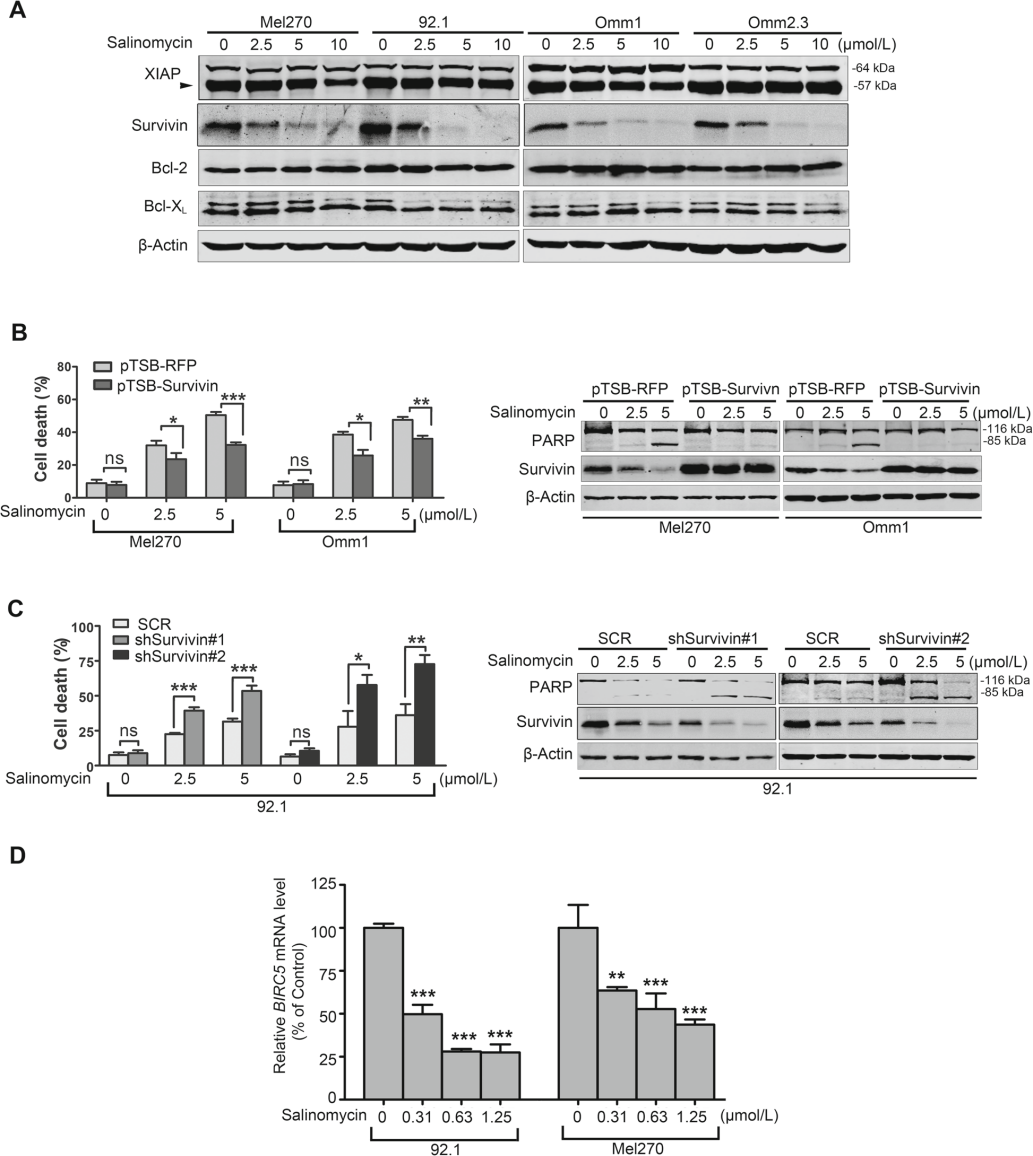
Survivin is essential for the salinomycin-induced apoptosis in UM cells. **a** UM cells were treated with salinomycin for 24 h and the protein levels of apoptosis-related proteins were detected by Western blot. **b** and **c** UM cells were transduced with lentiviral pTSB-Survivin cDNA **(b)**, Survivin-shRNA constructs **(c)**, or their corresponding empty vectors, and then incubated in the presence of puromycin (1 μg/mL) for 5 days to reach stable clones. Such survivin-manipulated cells were then exposed to salinomycin for 24 h, and subjected to trypan blue exclusion assay (*left*) and Western blot assay (*right*). Data represent mean ± SD. ns, not significant; *, *P* < 0.05; **, *P* < 0.01; ***, *P* < 0.001, Student’s *t* test. **d** qRT-PCR analysis of *BIRC5* mRNA level was done in the 92.1 and Mel270 cells treated with salinomycin for 24 h. **, *P* < 0.01; ***, *P* < 0.001, one-way ANOVA, post hoc comparisons, Tukey’s test

To unravel the mechanism of salinomycin-induced apoptosis in UM cells, we detected the expression of apoptosis-related proteins. Western blot results showed that salinomycin barely affected the protein level of apoptosis-related family members (XIAP, Bcl-2, and Bcl-X_L_) but Survivin (Fig. 3a). As it has been showed that the protein level of Survivin was highly expressed in 92.1 cells and lesser expressed in Mel270 and Omm1 cells [35], to functionally characterize the role of Survivin in apoptosis of UM cells in response to salinomycin, Mel270 and Omm1 cells were transduced with lentiviral construct encoding human *BIRC5* and establish stable clones, and then treated with or without salinomycin for 24 h. The transfection efficiency of Mel270, Omm1 and 92.1 cells was 80.0%, 87.0% and 89.6%, respectively (Additional file 1: Figure S1A and B). The knockdown efficiency of shSruvivin#1 and shSurvivin#2 was 63% and 68%, respectively (Additional file 1: Figure S1C). The data showed that salinomycin-enabled apoptosis was markedly crippled by ectopic overexpression of Survivin (Fig. 3b), but enhanced by knockdown of Survivin-shRNA as reflected by the cell death assayed by trypan blue exclusion and the specific cleavage of PARP in 92.1 cells (Fig. 3c).

To gain insight into the mechanisms that salinomycin decreases Survivin, we first examined Survivin turnover rate in presence or absence of salinomycin, and found that salinomycin displayed negligible effects on the turnover rate of Survivin (data not shown). However, qRT-PCR analysis showed that salinomycin significantly depressed the mRNA level of Survivin (Fig. 3d), suggesting that salinomycin decreases Survivin at the transcriptional level.

### Salinomycin dampens outgrowth of xenografted UM cells-derived tumors in NOD/SCID mice

**Fig. 4:**
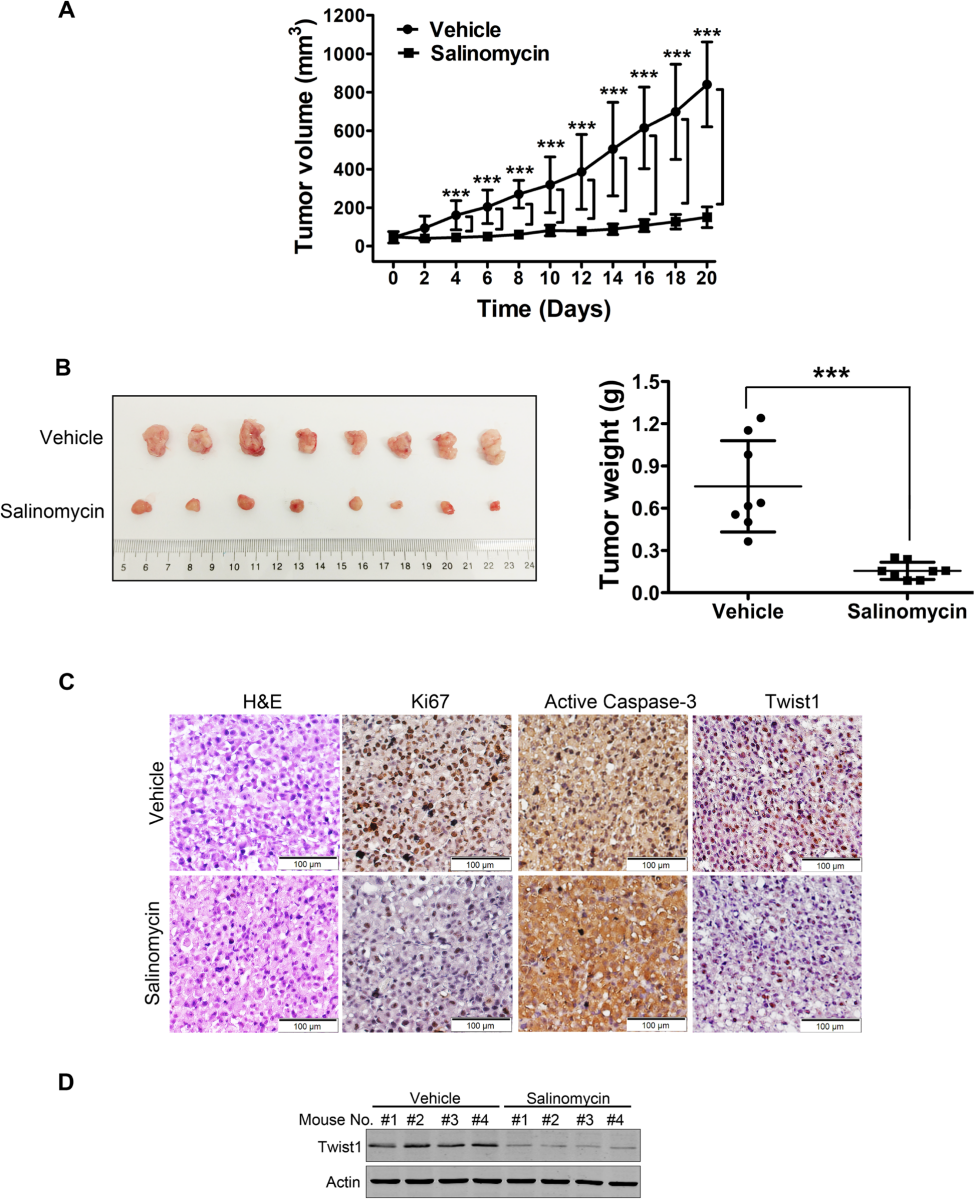
Salinomycin suppresses outgrowth of xenografted UM cells in NOD/SCID mice. **a** Tumor growth curve over time was plotted in the NOD/SCID mice injected (*i.p.*) with either vehicle (corn oil) or 2.5 mg/kg salinomycin daily. Data represent mean ± SD. ***, *P* < 0.001. Student’s *t* test. **b** Weights of tumors dissected on day 21 after administration with vehicle or salinomycin. Representative tumors are shown (*left*). Data (*right*) are the mean ± SD of tumor weights from each group. Vehicle (*n* = 8), salinomycin (n = 8). ***, *P* < 0.001. Student’s *t* test. **c** Hematoxylin & eosin (H&E) and immunohistochemistry (IHC) staining of Ki67, active caspase-3 and Twist1 in tumor tissue sections were conducted. Scale bar: 100 μm. **d** Protein levels of Twist1 from the tumors in NOD/SCID mice were analyzed with Western blot

We subsequently ascertained the antineoplastic effects of salinomycin in the NOD/SCID mice bearing xenografts of Omm1 cells. We found that both tumor volume (Fig. 4a) and tumor weight (Fig. 4b) were appreciably diminished in the mice administrated with salinomycin (2.5 mg/kg, *i.p.*, QD) for 21 days, as compared with mice of the corresponding vehicle-treated group. Importantly, no significant loss of body weight was observed in the salinomycin treatment group (data not shown). The immunohistochemistry (IHC) staining assays further confirmed the remarkable decrease of cell proliferation (marked as Ki67 staining) and increase of apoptosis (marked as active caspase-3 staining) in the xenografts upon salinomycin treatment (Fig. 4c).

### Salinomycin restricts migration and invasion of UM cells

**Fig. 5:**
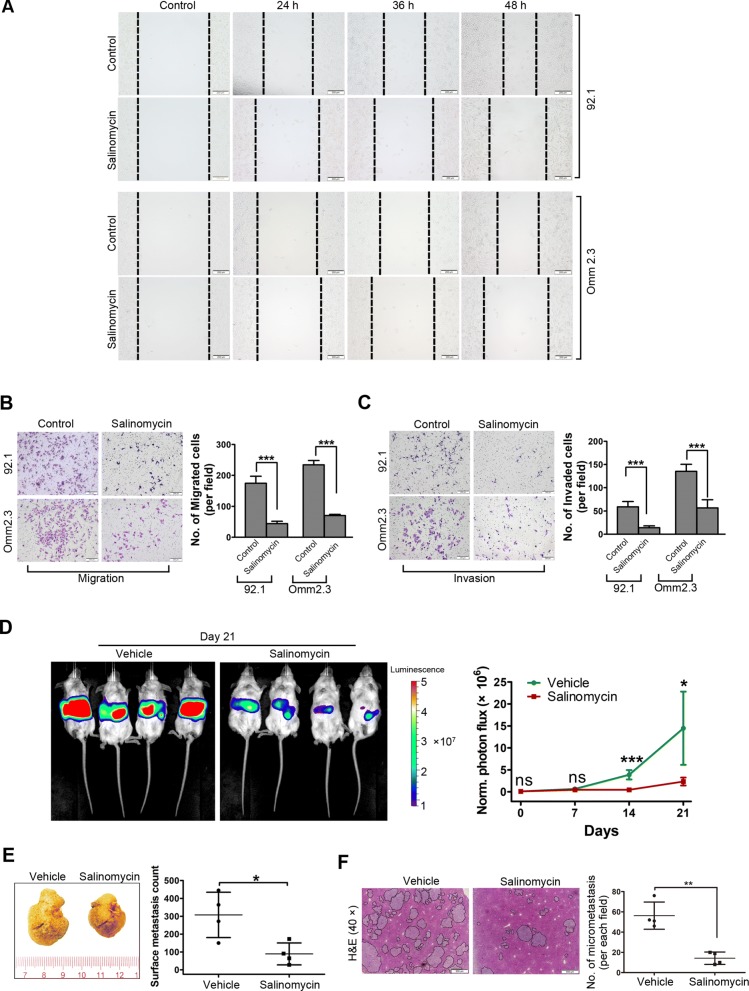
Salinomycin restrains hepatic metastasis in UM. **a** Photomicrograph of the wound healing scratch assay from control and salinomycin (1.25 μmol/L)-treated UM cells is shown. Scale bar: 200 μm. **b** and **c** Twenty-four hours after incubated with 1.25 μmol/L salinomycin, viable cells were counted and underwent transwell **(b)** or matrigel invasion chamber assays **(c)**. Representative images (*left*) for UM cells and quantitative analysis (*right*) from three random microscopic fields are shown. Data represent mean ± SD. ***, *P* < 0.001, Student’s *t* test. Scale bar: 200 μm. **d** After intrasplenic injection of 5 × 10^5^ of Mel270-Luc cells, the NOG mice were administrated vehicle (corn oil) or salinomycin (*i.p.*, 2.5 mg/kg, QD) every day for 21 days. In vivo bioluminescence imaging of hepatic metastasis after administration with salinomycin. *Left*, representative images of luciferase signals on day 21 after administration with vehicle (*n* = 4) or salinomycin (n = 4). *Right*, quantification of photon flux for hepatic metastases in NOG mice was performed every week. Data represent mean ± SD. ns, not significant; *, *P* < 0.05; ***, *P* < 0.001, Student’s *t* test. **e** Photomacrograph of liver was taken from the mice received intrasplenic injection of 5 × 10^5^ of Mel270-Luc cells and administration with vehicle or salinomycin for 21 days. *Right*, representative liver images are shown. *Left*, surface metastatic nodules in the liver from each group were counted. Data represent mean ± SD. *, *P* < 0.05, Student’s *t* test. **f** H&E staining in liver tissue sections. Scale bar: 500 μm. *Right*, representative H&E staining images in liver tissue sections are shown. *Left*, the number of micrometastasis in microscopic fields of each mice were counted. Data shown are the mean ± SD of number of micrometastasis in microscopic fields from 4 mice. **, *P* < 0.01, Student’s *t* test

Hepatic metastasis is a major malignant feature of UM and remains the leading cause of death in patients with UM [9]. We assessed the effects of salinomycin on migration and invasion of UM cells in vitro. As shown in Fig. 5a, wound healing scratch test of 92.1 and Omm2.3 cells showed a significant reduction in cell migration in response to salinomycin treatment. Analogously, in the transwell assay, much less UM cells migrated into the bottom chamber compared with that in the control (Fig. 5b). Moreover, the invasiveness of UM cells was considerably declined in the salinomycin-treated group as assessed by using the matrigel-coated transwell chamber assay (Fig. 5c). Taken together, these findings reveal that salinomycin exerts a drastically suppressive activity against migration and invasion in UM cells.

### Salinomycin obviates in vivo hepatic metastasis of UM

An intrasplenic transplantation liver metastasis model by intrasplenic injection of Mel270-Luc cells in NOG mice was next employed to evaluate the effect of salinomycin on UM metastasis in vivo. The results showed that the mice treated with salinomycin underwent marked bioluminescence signal retardation in liver zone (Fig. 5d), as well as diminished number of metastatic tumor nodules on the surface of livers (Fig. 5e). Consistently, H&E staining analysis also showed a robustly decline in size and number of metastatic foci on the livers of salinomycin-treated mice relative to the vehicle-treated mice (Fig. 5f). These experimental results support the notion that salinomycin can markedly abrogate the hepatic metastasis of UM cells in vivo.

### Salinomycin decreases cancer stem-like cells in UM

**Fig. 6:**
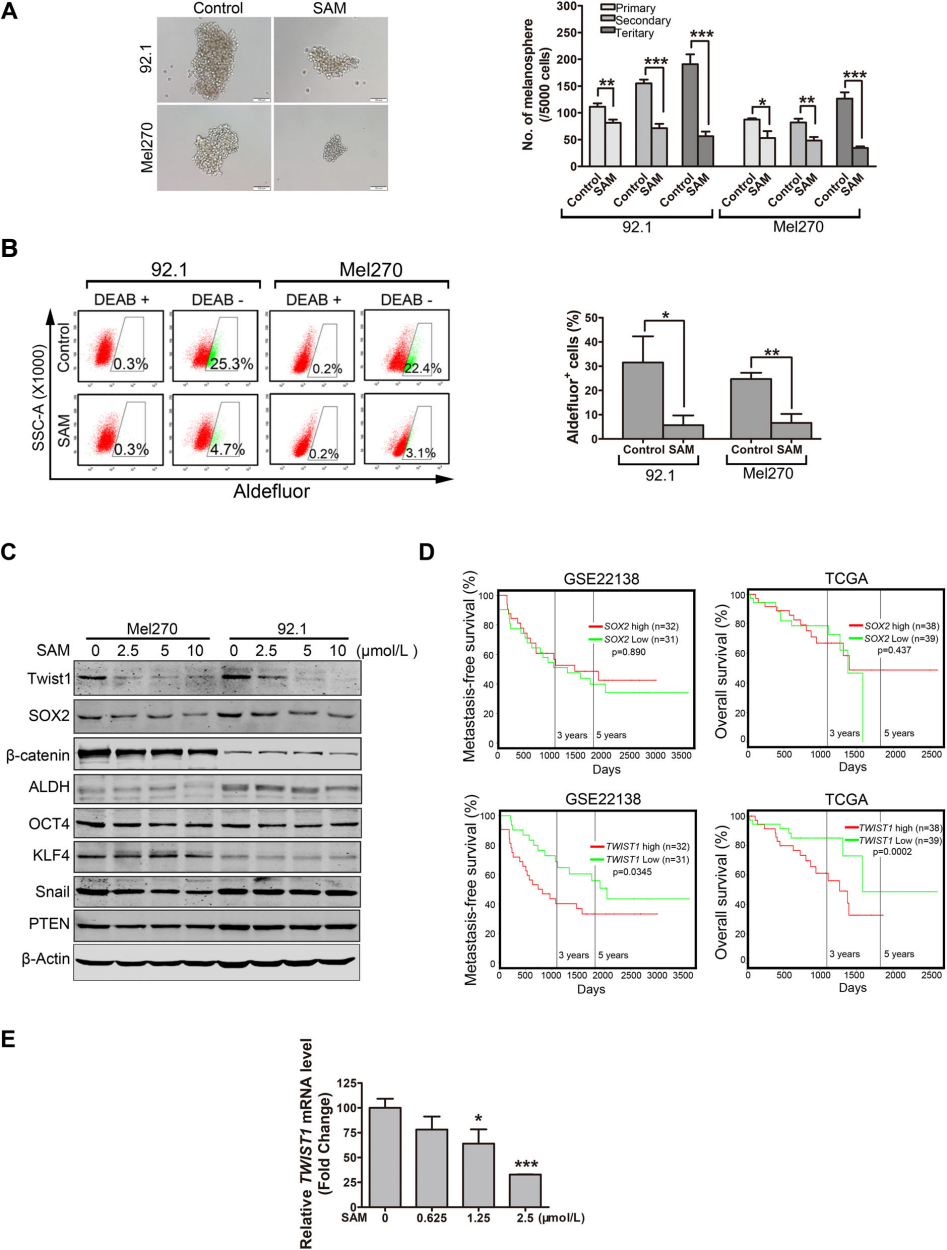
Treatment with salinomycin restricts the self-renewal potential and reduces the Aldefluor^+^ cells in UM cells. **a** Twenty-four hours after Mel270 and 92.1 cells were treated with SAM (salinomycin) (1.25 μmol/L), 5000 viable cells resuspended in melanosphere culture medium were seeded into the ultralow- attachment 24-well plates. Melanospheres were counted and photographed on day 7. The cells were then harvested and re-plated (5000 cells/well) for the secondary and tertiary rounds of melanosphere culture, respectively. *Left*: representative images of melanospheres. Scale bar: 100 μm. *Right:* quantification of melanospheres. Data represent mean ± SD. *, *P* < 0.05; **, *P* < 0.01; ***, *P* < 0.001, one-way ANOVA, post hoc comparisons, Tukey’s test. **b** Mel270 and 92.1 cells were incubated with 1.25 μmol/L salinomycin for 24 h, Aldefluor^+^ cells were measured by using a FACS LSRFortessa flow cytometer. Representative flow cytometry dot plots (*left*) for UM cells and quantitative analysis (*right*) from three independent experiments are shown. Data represent mean ± SD. ns, not significant; *, *P* < 0.05; ***, *P* < 0.001, Student’s *t* test. **c** 92.1 and Mel270 cells were treated with salinomycin for 24 h, the protein levels of stemness-related proteins in control or salinomycin- treated cells were determined by Western blot. **d** Kaplan–Meier survival analysis of liver metastasis-free survival and overall survival of patients with UM was done based on the median expression level of mRNA (*SOX2* or *TWIST1*) from GSE22138 dataset and TCGA dataset, respectively. **e** qRT-PCR analysis of *TWIST1* mRNA level after treatment with salinomycin in 92.1 cells. *, *P* < 0.05; ***, *P* < 0.001, one-way ANOVA, post hoc comparisons, Tukey’s test

Given that salinomycin has been identified as a selective inhibitor of CSCs in multiple cancers [17–19], we next assessed whether salinomycin had the capacity to eliminate CSCs in UM. We first examined the self-renewal capacity by using melanosphere formation and serially re-plating assay. The results showed that not only the size and number of melanospheres but also the re-plating capacity were significantly diminished in the salinomycin-treated UM cells (Fig. 6a). In addition, the results showed that salinomycin dramatically reduced the percentage of Aldefluor positive cells in 92.1 and Mel270 (Fig. 6b). These results also reveal that salinomycin is active against CSCs in UM cells.

In order to illuminate the underlying mechanism that salinomycin effectively suppresses the phenotypes of CSCs, we detected the protein levels of intrinsic regulators including stemness-related transcriptional factors. The results showed that the levels of Twist1 and SOX2 were lowered in salinomycin-treated 92.1 and Mel270 cells (Fig. 6c). Further analysis with two sets of publicly available GSE22138 and TCGA database unveiled that the mRNA levels of *TWIST1* rather than *SOX2* conversely correlated with the metastasis-free survival and overall survival in patients with UM (Fig. 6d). Additionally, the IHC staining assays and Western blot assay further confirmed the Twist1 proteins were decreased in the tumor xenografts of the salinomycin-treated mice (Fig. 4c and d). Moreover, the salinomycin dose-dependently diminished TWIST1 transcription as evaluated by qRT-PCR in 92.1 cells (Fig. 6e). These data indicate that increased *TWIST1* may confer the aggressive features of UM cells. We therefore paid our attention to TWIST1 in the subsequent experiments to evaluate the deleterious effect salinomycin on CSCs.

### Salinomycin-mediated depletion of Twist1 is essential for elimination of CSCs in UM

**Fig. 7:**
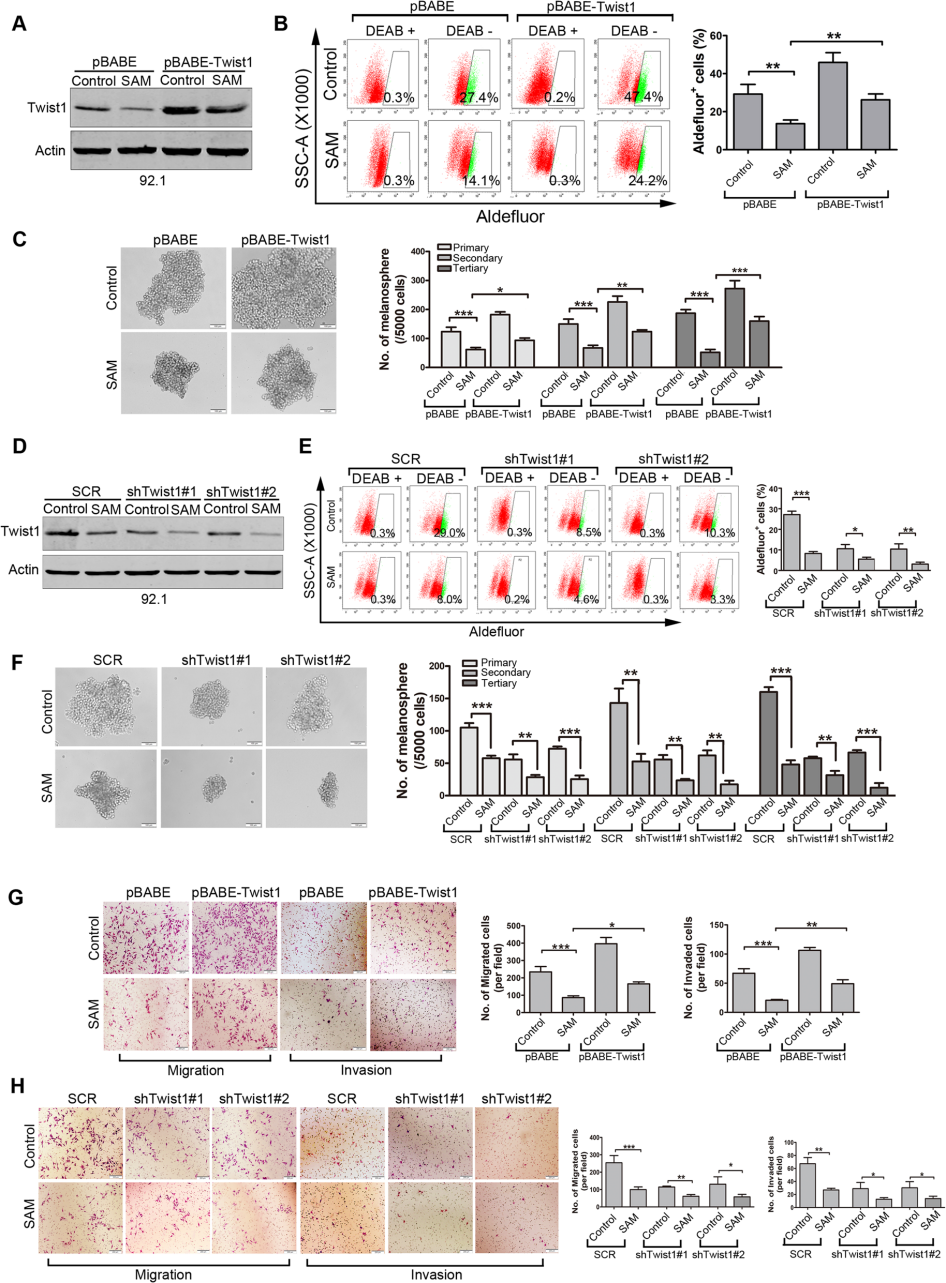
Salinomycin impedes traits of CSCs at least partially through suppressing Twist1. **a-c** 92.1 cells transduced with retroviral Twist1 cDNA-expressing constructs were incubated in the presence or absence of salinomycin for 24 h, and then subjected to assays of Western blot **(a)**, Aldefluor assay **(b)** and melanosphere **(c)**, respectively. Representative images for UM cells and quantitative analysis from three independent experiments are shown. Data represent mean ± SD. *, *P* < 0.05; **, *P* < 0.01; ***, *P* < 0.001; one-way ANOVA, post hoc comparisons, Tukey’s test. **d-f** 92.1 cells stably transduced with lentiviral Twist1-shRNA constructs were treated with or without 2.5 μmol/L salinomycin for 24 h, and then subjected to Western blot **(d)**, Aldefluor assay **(e)**, melanosphere assays **(f)**, respectively. Representative images for UM cells and quantitative analysis from three independent experiments are shown. Data represent mean ± SD. *, *P* < 0.05; **, *P* < 0.01; ***, *P* < 0.001; Student’s *t* test. **g and h** UM cells transduced with retroviral constructs of Twist1-encoding or lentiviral shRNA against Twist1 were exposed to 2.5 μmol/L salinomycin for 24 h, then underwent transwell migration and matrigel invasion assay. *, *P* < 0.05; **, *P* < 0.01; ***, *P* < 0.001; one-way ANOVA, post hoc comparisons, Tukey’s test for g and Student's t test for h

To further explore the role of Twist1 in UM cells, 92.1 cells transduced with retroviral Twist1-encoding constructs were incubated in presence or absence of 2.5 μmol/L salinomycin for 24 h (Fig. 7a), and then subjected to flow cytometry for ALDH^+^ cells and melanosphere assay. The results showed that forced expression of Twist1 increased the proportion of Aldefluor^+^ cells (Fig. 7b) as well as melanosphere formation (Fig. 7c) in 92.1 cells. Ectopic expression of Twist1 attenuated the salinomycin-induced reduction in Aldefluor^+^ cells percentage and serial melanosphere formation capacity (Fig. 7b and c). Conversely, silencing Twist1 by lentiviral shRNA (Fig. 7d) reduced the percentage of Aldefluor^+^ cells (Fig. 7e) and crippled the capacity of melanosphere formation in 92.1 cells (Fig. 7f). Silencing Twist1 by lentiviral shRNA potentiated the salinomycin-induced reduction in Aldefluor^+^ cells percentage and serial melanosphere formation capacity (Fig. 7e and f).

### Salinomycin-mediated depletion of Twist1 facilitates reduction in migration and invasion of UM cells

Given that Twist1 can promote invasive properties of cancer cells [36], we next examined the role of Twist1 in salinomycin-mediated blockage in migration and invasion. In line with previous report, we observed that ectopic expression of Twsit1 promoted, while knockdown of Twist1 decreased the migration and invasion of 92.1 cells (Fig. 7g and h). Furthermore, the salinomycin-attenuated migration and invasion was considerably reversed by forced expression of Twist1, but potentiated by knockdown of Twsit1 by shRNA (Fig. 7g and h). Taken together, these results imply that Twsit1 is fundamental for salinomycin-enabled CSCs phenotype inhibition in UM cells.

## Discussion

In the present study, we revealed that salinomycin showed a remarkably inhibitory effect on growth and survival in vitro and in vivo in UM cells. We also found that Survivin is critical for the salinomycin-enabled apoptosis. Meanwhile, we demonstrated that salinomycin displayed potently inhibitory effects on UM CSCs and efficiently suppresses migration and invasion in vitro and significantly hampers hepatic metastasis in vivo. Additionally, our data suggest that Twist1 at least partially contributes to the onset of salinomycin-enabled CSCs elimination and migration/invasion restriction in UM.

Salinomycin, a monocarboxylic polyether antibiotic, has been safely used as a coccidiostatic antibiotic and growth promoter in livestock for decades [21, 37]. However, for considerable toxicity of salinomycin in mammals, it has not been applied to human until the discovery of its antineoplastic activity. Surprisingly, a small scale of clinical trial has demonstrated that salinomycin only exerted minimal side effects at antineoplastic dosages (200–250 μg·kg^− 1^, *i.v.*, every second day for 3 weeks) in human [21]. Analogously, our data also showed that the cytotoxicity of salinomycin on the UM cells is about 11~21-fold as high as that of the normal retinal pigmented epithelium cells, which implied the safety of salinomycin used in treatment of UM.

Colonization of tumor cells in target organ is a critical step for formation of metastases [11]. Our results demonstrated that salinomycin potently inhibited proliferation of UM cells in vitro and in NOD/SCID mice. In concordance with our observations in UM cells, the inhibitory effect of salinomycin on proliferation has been also found in other types of cancer such as breast cancer and pancreatic cancer [17, 37]. Mechanistically, Survivin plays a role for salinomycin-induced apoptosis, which is consistent with the findings observed in the breast cancer and ovarian cancers [38, 39]. Obviously, induction of apoptosis by salinomycin may confer inhibition of metastasis in UM cells.

In the UM cells, studies have demonstrated that stemness-associated factors (e.g., Twist1, Zeb1, and Snail1) have the capacity to facilitate invasive properties [27, 36]. Twist1, a member of the bHLH (basic helix-loop-helix) transcription-factor family, boosts stemness by transcription repression of E-cadherin [40, 41]. Consistent with previous report [36], we found that high expression of Twist1 was correlated with poor metastasis-free survival and overall survival by analyzing of the publicly available database in UM. These data reveal that targeting Twsit1 may provide a promising approach for treatment of UM patients with hepatic metastasis. Additionally, we discovered that salinomycin can effectively induce transcription repression of Twist1, implicating a deepening explanation for salinomycin-mediated elimination of CSCs. It will be worthy to test in future in other types of cancer.

Given that Twist1 only partially reversed the salinomycin-enabled CSCs eradiation and hepatic metastasis obviation in UM cells, other regulators may also involve in the salinomycin-mediated effect on UM cells. Of note, salinomycin significantly reduced the protein level of SOX2. Considering the crucial role of SOX2 in the maintenance of stemness of CSCs in multiple types of solid tumors [42–44], the possibility of salinomycin-enabled reduction of SOX2 in the elimination of CSCs in UM cannot be totally excluded although high expression of SOX2 was not correlated with metastasis-free survival.

## Conclusions

In summary, salinomycin has considerable inhibitory effect on UM in vitro and in vivo. Salinomycin induced apoptosis in UM cells at least partially through repressing Survivin. Moreover, salinomycin efficiently depresses the transcription of Twist1 which is fundamental for the salinomycin-mediated CSCs eradiation and migration/invasion restriction in UM cells. Our results shed light on the molecular mechanism of antineoplastic activity of salinomycin and demonstrated that salinomycin may be a potential agent to eliminate UM CSCs and to impede hepatic metastasis in UM. These findings warrant a clinical trial of salinomycin in UM patients with hepatic metastasis.

## Supplementary information

**Additional file 1:**The transfection and knockdown efficiency of UM cells.

## Data Availability

Not applicable.

## Abbreviations

ALDH: aldehyde dehydrogenase
bHLH: basic helix-loop-helix
BIRC5: baculoviral IAP repeat containing 5
BSA: bovine serum albumin
CSCs: cancer stem-like cells
EMT: epithelial-mesenchymal transition
GPCR: G protein coupled receptor
H&E: hematoxylin & eosin
IHC: immunohistochemistry
MAPK: mitogen-activated protein kinase
PARP: poly (ADP-ribose) polymerase
PBS: phosphate-buffered saline
PI: propidium iodide
PKC: protein kinase C
SAM: salinomycin
UM: uveal melanoma

## Supplementary information

**Supplementary information** 10.1186/s12943-019-1068-1
