# Correction to: Salinomycin effectively eliminates cancer stem-like cells and obviates hepatic metastasis in uveal melanoma

**DOI:** 10.1186/s12943-021-01334-6

**Published:** 2021-03-03

**Authors:** Jingfeng Zhou, Shenglan Liu, Yun Wang, Wei Dai, Hailin Zou, Shubo Wang, Jing Zhang, Jingxuan Pan

**Affiliations:** State Key Laboratory of Ophthalmology, Zhongshan Ophthalmic Center, Sun Yat-sen University, 54 South Xianlie Road, Guangzhou, 510060 People’s Republic of China

**Correction to: Mol Cancer 18, 159 (2019).**

**https://doi.org/10.1186/s12943-019-1068-1**

After publication of the article [1], the authors realized one misplaced image in Fig. 2a and Fig. 2d, respectively, which prompted us to request correction. These errors inadvertently happened during the stage of our figure assembly with photoshop microsoftware. The corrected version of Fig. 2a and Fig. 2d is provided. The correction does not affect the conclusion or discussion of this article. The authors deeply apologize to readers the inconvenience caused by the unconscious mistakes and appreciate the correction opportunity.

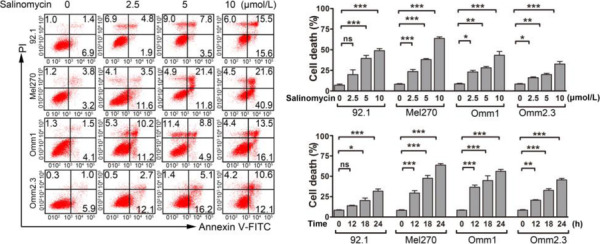



Fig. 2 **a**. Annexin V/PI apoptotic assay was performed in the UM cells treated with escalating concentrations of salinomycin for 24 h or at 10 μmol/L for various exposure time. Representative flow cytometry dot plots (*left*) for UM cells and quantitative analysis (*right*) from three independent experiments are shown. Data represent mean ± SD. ns, not significant; *, *P* < 0.05; **, *P* < 0.01; ***, *P* < 0.001, one-way ANOVA, post hoc comparisons, Tukey’s test

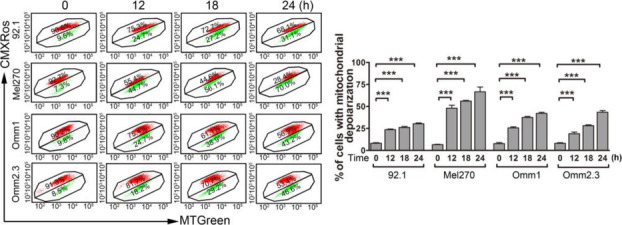



Fig. 2 **d**. UM cells were treated with 10 μmol/L salinomycin for the time indicated, and the mitochondrial potential was then detected by flow cytometry after dual staining with CMXRos and MTGreen. Data represent mean ± SD. ***, *P* < 0.001, one-way ANOVA, post hoc comparisons, Tukey’s test

## References

[1] Zhou J, Liu S, Wang Y, Dai W, Zou H, Wang S, Zhang J, Pan J (2019). Salinomycin effectively eliminates cancer stem-like cells and obviates hepatic metastasis in uveal melanoma. Mol Cancer.

