# Supplementary figures and images for: Salinomycin effectively eliminates cancer stem-like cells and obviates hepatic metastasis in uveal melanoma
Source: Mol Cancer. 2019 Nov 13;18:159. doi: 10.1186/s12943-019-1068-1 (PMC6852970; doi:10.1186/s12943-019-1068-1)

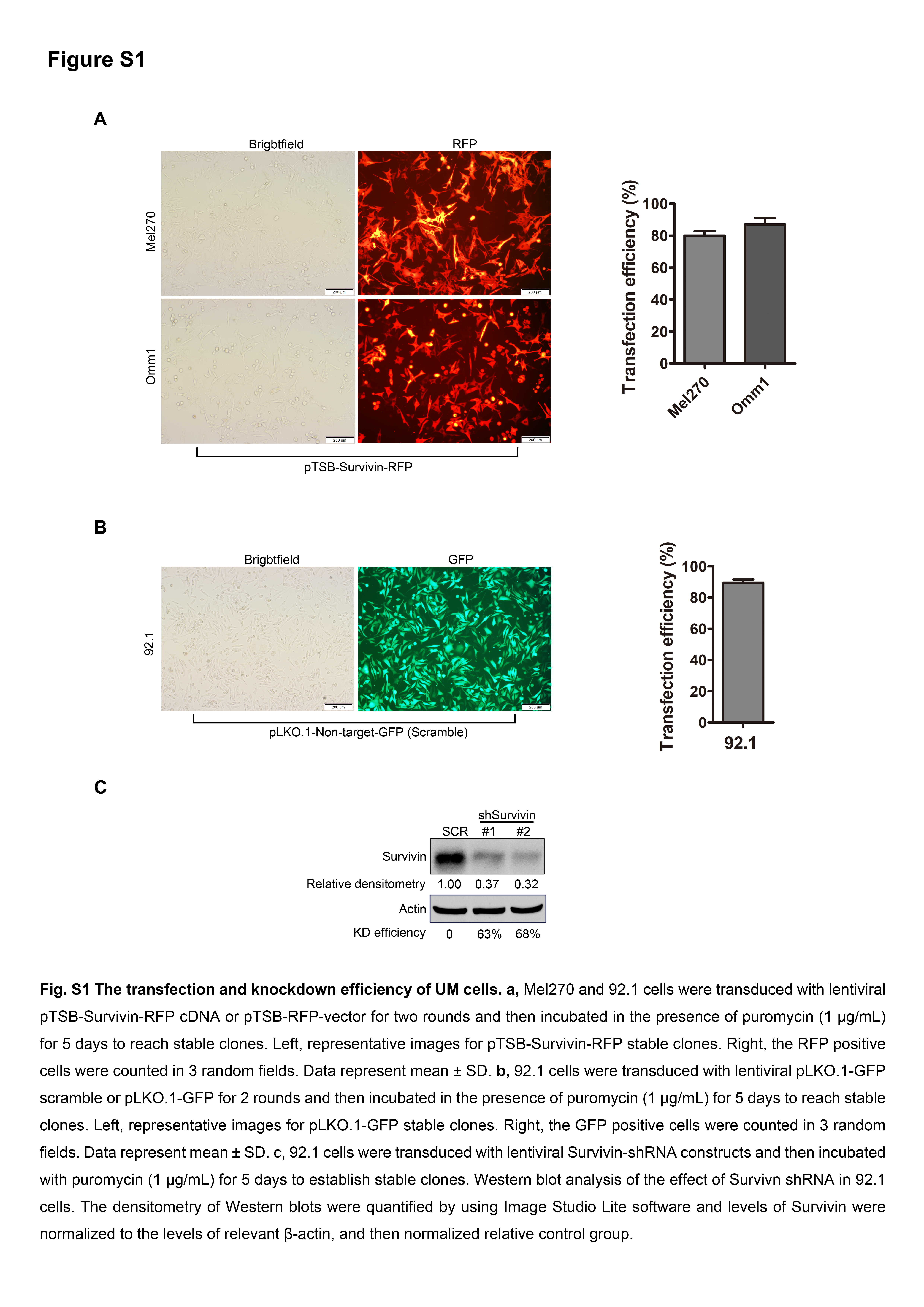

Supplement: Supplementary file 1 — Additional file 1:The transfection and knockdown efficiency of UM cells. [file 12943_2019_1068_MOESM1_ESM.tif]
